# Evaluation of the efficacy of optimal pulsed technology treatment in patients with cataract and Meibomian gland dysfunction in the perioperative period

**DOI:** 10.1186/s12886-020-01357-5

**Published:** 2020-03-18

**Authors:** Jinling Ge, Na Liu, Xiaoming Wang, Ying Du, Chaoqing Wang, Zhaorui Li, Jing Li, Lihua Wang

**Affiliations:** 1 Department of Ophthalmology, Jinan Mingshui Eye Hospital, Jinan, Shandong Province China; 2 Department of Ophthalmology, Provincial Hospital, Shandong University, NO. 324 Jingwuweiqi Road, Jinan, Shandong Province China

**Keywords:** Age-related cataract, Phacoemulsification, Dry eye, Meibomian gland dysfunction, OPT treatment

## Abstract

**Background:**

The aim of this study was to evaluate the efficacy and safety of M22 Optimal Pulsed Technology (OPT) applied in patients with age-related cataract and Meibomian gland dysfunction (MGD) in perioperative period.

**Methods:**

This prospective observational study was carried out in the Jinan Mingshui Eye Hospital (Zhangqiu, China). We studied 60 patients (30 in the OPT treatment group and 30 in the conventional surgery group) with age-related cataract and MGD who underwent phacoemulsification and evaluated the efficacy of OPT treatment before and 1 month and 3 months after surgery. Ocular Surface Disease Index (OSDI) questionnaire, biomicroscopic examination of lid margins, Meibomian gland yielding secretion score (MGYSS), corneal fluorescein staining scores (CFS), tear film break-up time (TBUT), tear meniscus height (TMH) and the morphology of the MG (meibography) followed by Keratograph 5 M (K5M) were used to assess the patients’ conditions.

**Results:**

There were significant differences in the scores of OSDI, MGYSS, TBUT, and CFS between the preoperative and postoperative outcomes (*p* < 0.05). In the OPT treatment group, the postoperative ocular surface condition was obviously better and the patient satisfaction rate was higher than those before surgery. There were significant differences in the scores of OSDI, EMAS, MGYSS and CFS before and 1 month after surgery (*p* < 0.05). In addition, there were also significant differences in the scores of OSDI, EMAS, MGYSS and MGLS before and 3 months after surgery (*p* < 0.05). No complications appeared during OPT treatment.

**Conclusions:**

Cataract surgery can aggravate MGD and is detrimental to ocular surface health. OPT treatment was a safe and effective intervention for patients with MGD and cataract during perioperative period.

## Background

Age-related cataract (ARC) is one of the most important causes of visual impairment in the world [1]. With the trend of population ageing, ARC will become the most common eye disease in the world in 2020 [2]. Cataract surgery is one of the most common procedures performed worldwide, and excellent postoperative visual acuity is usually obtained [3]. However, dry eye syndrome (DES) usually occurs after cataract surgery. DES is an ocular surface disease caused by a variety of reasons, characterized by loss of steady state of the tear film and dry eye symptoms, and its pathogenesis includes corneal nerve injury, ocular surface inflammation, goblet cell decrease, and Meibomian gland dysfunction (MGD) [4].

MGD is a chronic diffuse abnormality of the meibomian glands, usually characterized by terminal duct obstruction and changes in glandular secretion [5]. The prevalence of MGD is as high as 70% in Asians, which has attracted wide attention of clinicians and scientists [6, 7]. MGD can increase tear evaporation and tear osmotic pressure and lead to inflammation of the corneal surface and damage to the corneal epithelium. Therefore, MGD is the major cause of evaporative dry eye and contributes to aqueous-deficient dry eye. Dry eye and tear film dysfunction after cataract surgery, including foreign body sensation, burning sensation, itchy eyes, dryness, poor near sight, redness, decreased contrast sensitivity and irritation, are closely related to MGD [8–14].

There are many clinical treatments for MDG, including artificial tears, warm compression, meibomian gland expression, omega-3 supplementation, cyclosporine, corticosteroids and oral antibiotics. However, those treatment methods have been shown to provide short-term relief of symptoms [15]. In-tense pulsed light (IPL) treatment applies Xenon flash lamp to emitting wavelengths of light ranging from 590 to 1200 nm, which has been used in treatment of rosacea, elangiec-tasia, port-wine stains, and pigmentation of the skin around the eyes. In recent years, IPL has been extended to treat MGD, and has also been introduced into DEWS II [16, 17]. Optimized Pulse Technology (OPT) is adopted in the M22 system (Lumenis Medical Laser Co. Ltd., Yokneam, USA). Its square wave pulse shape is uniform, and the time and energy of intense light emission are more accurate, safe and effective. The three-pulse square wave without energy spikes and attenuation has the advantage of rapidly increasing the temperature of the target tissue under the epidermis to achieve its destruction, while maintaining skin integrity. The sapphire contact cooling technology allows the patient to feel more comfortable and pain free during treatment.

The aim of our study was to evaluate the effect of OPT therapy on patients with age-related cataract combined with mild to moderate MGD. In this study, ocular surface disease index (OSDI) questionnaire, eyelid margin abnormality score (EMAS), Meibomian gland yielding secretion score (MGYSS), corneal fluorescein staining (CFS) scores and Keratograph 5 M (K5M) examination for patients before and after surgery were analyzed to evaluate the effects of OPT therapy on postoperative functional symptomology in patients with MGD combined with age-related cataract.

## Methods

### Patients

This study was a prospective observational study, and was conducted in accordance with the ethical guidelines of the Declaration of Helsinki and was approved by the Human Research and Ethics Committee of Jinan Mingshui Eye Hospital (Jinan, China) (No. 20170802). Written informed consent was obtained from each participant before enrolment. A total of 60 patients with AGC who had mild to moderate MGD in the Mingshui Eye Hospital from October 2017 to December 2017 were included in this study. There were 30 patients with mild MGD and 30 patients with moderate MGD.

Inclusion criteria: (1) patients who were diagnosed as age-related cataract and eligible for cataract surgery; (2) according to the consensus of experts in the diagnosis and treatment of MGD in China in 2017 **(**Table 1**)** [8], patients who were diagnosed as mild to moderate MGD; (3) patients who had no history of diabetes, hypertension, and systemic autoimmune diseases such as Sjögren syndrome; (4) Fitzpatrick [18] Skin Classification Type was 1–4; (5) patients who had good education level and normal communication skill, and could communicate with the researchers and express their treatment experience; (6) patients who could understand the different treatment options and volunteer to participate in the study.

**Table 1:** The graduation standard of MGD

Degree	Symptoms	Palpebral margin changes	Secretion character score	Secretion discharge capacity score	Meibomian gland deletion score	Corneal
Mild	Slight, intermittent	Normal or mild hyperemia of palpebral margin and there may be fat cap formation	1	1	1	Normal, no epithelial damage
Moderate	Mild or moderate, persistent	palpebral margin becomes blunt, round and thickened. Meibomian gland mouth was obstruction and protuberance	2	2	2	Mild or moderate epithelial damage, located at the periphery
Severe	Moderate or severe, affecting life or work	The blepharon margin is thickened and the neovascularization is obvious. Fat thrombus formation in meibomian gland mouth	3	3	3	Damage to epithelium and superficial matrix

Exclusion criteria: (1) patient with infectious blepharitis, seborrheic blepharitis and rouge high-emission MGD; (2) patients had history of ocular trauma or surgery or long-term medication; (3) patients with severe ocular surface abnormalities; (4) patients had obvious abnormalities in the eyelid margins (> 3 times of positive surgery), reduced meibum expression (grade > 2) or obstructed gland dropout (meibography score > 3).

### Evaluation of MGD and DE parameters

The parameters of MGD were assessed by the consensus of the experts on the diagnosis and treatment of meibomian gland dysfunction in 2017 **(**Table 1**)** [8]. Each patient underwent routine ophthalmologic examinations, including naked eye and corrected visual acuity, intraocular pressure, slit lamp microscopy (eyelid margin abnormality score, meibomian gland yielding secretion score, and corneal fluorescein staining) and fundus examination. After 30 min of rest, DE questionnaire and DE related examination were performed in the order of OSDI questionnaire, tear meniscus height (TMH), tear break-up time (TBUT) and (MGLS). Examinations of TMH, TBUT, and MG were performed using a K5M ocular surface analyzer. All MGD-related examinations were required to be completed before using eye drops (antibiotic eye drops and topical anesthetics).

### Preoperative evaluation of cataract

All patients completed the OSDI questionnaire, which was scored according to previous describes [19]. The 12 items of the OSDI questionnaire were graded on scale 0 to 4, of which 0 indicated no time; 1, sometimes; 2, half of the time; 3, most of the time; and 4, all of the time. The total OSDI score was then calculated on the basis of the following formula: OSDI = [(sum of scores for all questions answered) × 100]/ [(total number of questions answered) × 4]. Thus, the OSDI score was based on a 0 to 100 scale and higher scores indicated more severe symptoms or discomfort.

Microscopic examination. (1) Eyelid margin abnormality score (EMAS) [20]: Eyelid margin abnormalities were scored as 0 (absent) or 1 (present) for the following 4 parameters: vascular engorgement, plugged meibomian gland orifices, anterior or posterior displacement of the mucocutaneous junction, and irregularity of lid margin. The sum was recorded as 0 through 4. (2) Meibomian gland yielding secretion score (MGYSS). The quality degree of the meibum was based on the following: grade 0, clear; grade 1, cloudy; grade 2, cloudy with granular debris; and grade 3, thick like tooth-paste. The upper and lower eyelids of each eye were scored separately, 0 was normal, 1 point and above were abnormal, and the highest score of this item was 6 points. (3) Corneal fluorescein staining (CFS). The cornea was stained with 0.2% sodium fluorescein and positive staining indicated the integrity of the corneal epithelial cells. CFS used the 12-point method [21]: the cornea was divided into four quadrants, each quadrant was scored according to the following criteria: 0, no spot dyeing; 1, 1–30 spots dyeing; 2, > 30 spots dyeing but not fused into tablets; 3, corneal spots dyed point fusion or ulcers.

K5M ocular surface comprehensive analyzer inspection. All selected patients were inspected by the same technician under the operation of the K5M: TMH, TBUT and MGLS.

### Cataract surgery

A total of 60 patients were randomly divided into two groups: OPT treatment group and conventional surgery group. Conventional surgery group: patients were routinely prepared according to the clinical path of cataract surgery. OPT treatment group: in addition to routine preoperative preparation according to the clinical path of cataract surgery, the patients in OPT treatment group also received M22 OPT (OPT, Oculus, Wetzlar, K5M Germany) treatment before and 1 and 2 months (± 2 days) after surgery. OPT treatment was performed by the same skin cosmetic surgeon. The operation of OPT treatment was as follows: (1) Washed and dried the face; (2) The patients were asked to wear a special protective eye mask and close eyes; (3) Parameter design: the mode was three-pulse, the pulse time was 6 ms, the pulse interval was 50 ms, and the energy density was (11–16) J/cm^2^; (4) Ultrasound gel was applied on the patient’s face; (5) using 35 mm × 15 mm light guide crystal; (6) From the inferior temporal margin near the lateral malleolus to the nasal side, 12–16 laser spot were treated. (7) The wavelength of the filter was 590 nm. All cataract surgeries were performed by the same experienced surgeon [22]. The 2.2 mm three-plane tunnel incision on angle scleral was taken over the iliac crest.

No complications occurred during and after surgery. After the treatment, the specialist nurses carried out detailed health education for the patients and their families. Avoid hot water contact (such as sauna, steaming, hot bath, etc.) on the face within 48 h after treatment. Do not rub, scratch or make up. If there was scab in the local area, the scab would be removed within 1–2 weeks and the wound would be healed. Before removing the scab, the infection of the wound should be prevented, the wound should be kept dry, and the pigmentation should be prevented. Avoid direct sunlight exposure after treatment. The treatment area should be well hydrated and repaired. Usually, eye use time should not be too long. After cataract surgery, in addition to routine administration of antibiotics (such as Levofloxacin Eye Drops) and hormone eye drops (such as cortisone eye drops), the patients in OPT treatment group were received OPT treatments at 1 month and 2 months (±2 days) after surgery.

### Postoperative follow-up

The follow-up which was performed by the same ophthalmologist was performed for 1 month (the OPT treatment group was performed before the second OPT treatment) and 3 months after the operation in the following order: OSDI questionnaire, slit lamp examination (EMAS, MGYSS, and CFS), and K5M (Oculus, Wetzlar, Germany) examination (TMH, TBUT, and MGLS).

### Statistical analyses

Statistical analysis was performed using SPSS 23.0 (SPSS Inc., Chicago, IL, USA). Normal distribution of the data was verified by using the Kolmogorov-Smirnov test. The scores of OSDI, TMH and TBUT were normally distributed values and data were expressed as means ± SD. EMAS, MGYSS, CFS and MGLS were non-normally distributed values and data were expressed as Median (P25, P75). Continuous intergroup variables were analyzed by using an independent t-test, and continuous intragroup variables were tested by a paired t-test. Categorical intergroup variables were analyzed with the nonparametric Kruskal–Wallis test, and categorical variables intragroup were analyzed with the nonparametric Wilcoxon signed-rank test. *P* < 0.05 was considered to be statistically significant.

## Results

### General clinical symptoms

We studied 30 patients with AGE and MGD for OPT treatment in this study. 8 patients were lost to follow-up and the remaining 22 patients were the subjects of this group. The mean age of the 22 patients was 63.48 ± 8.47 years old (ranged from 56 to 79 years) and 12 patients were female. As for conventional surgery group, we evaluated 30 patients. 5 patients were lost to follow-up and the remaining 25 patients were the subjects of this group. The mean age of the 25 patients was 65.8 ± 8.1 years old (ranged from 54 to 84 years) and 14 patients were female. There were no significant differences in gender and age between the two groups (*p* > 0.05).

### Changes in DE syndrome and ocular surface parameters before and after cataract surgery in the conventional operation group

There were significant differences between OSDI_0_ (pre-OSDI score) and OSDI_1_ (OSDI score at 1 month postoperatively) (31.19 ± 7.28 vs 33.43 ± 6.32, *p* = 0.003) (Table 1). However, there was no significant difference between OSDI_0_ and OSDI_3_ (31.19 ± 7.28 vs 30.51 ± 6.65, *p* = 0.256) **(**Table 2**)**. It showed that the dry eye symptoms were significantly aggravated 1 month after the operation and recovered to preoperative levels 3 months after the operation.

**Table 2:** Comparison of dry eye symptoms and ocular surface parameters in the Conventional surgery group before and after surgery

Parameters	baseline	1 month	3 month	*p* value		
				baseline vs 1 month	baseline vs 3 month	1 month vs 3 month
OSDI^a^	31.19 ± 7.28	33.43 ± 6.32	30.51 ± 6.65	0.003*	0.256	0.001#
EMAS^b^	1.00 (1.00, 2.00)	1.00 (1.00, 2.00)	1.00 (1.00, 2.00)	0.109	0.334	0.763
MGYSS^b^	1.00 (1.00, 1.00)	1.00 (1.00, 2.00)	1.00 (1.00, 2.00)	0.088	0.002*	0.376
CFS^b^	0.00 (0.00, 1.00)	1.00 (0.50, 1.00)	0.00 (0.00, 1.00)	0.074	0.008*	0.564
TMH^a^/mm	0.18 ± 0.03	0.20 ± 0.02	0.20 ± 0.02	0.016*	0.020*	0.635
NITBUT^a^/s	5.52 ± 1.95	5.06 ± 1.54	4.99 ± 1.24	0.002*	0.035*	0.764
MGLS^b^	1.00 (1.00, 2.00)	1.00 (1.00, 1.50)	1.00 (1.00, 2.00)	0.564	0.655	0.157

*OSDI* Ocular Surface Disease Index, *MGYSS* Meibomian gland yielding secretion score, *CFS* corneal fluorescein staining, *TMH* tear meniscus height, *EMAS* Eyelid margin abnormality score, *MGLS* meibomian gland loss score, *TBUS* tear film break-up time. a: Normal distribution data, the mean is expressed as Mean ± SD, and the paired sample t test is used for comparison between groups. b: Non-normally distributed data, the mean is represented by Median (P25, P75), and the comparison between groups is based on paired sample nonparametric Wilcoxon test. *p < 0.05 vs Baseline; #*p* < 0.05 vs 1 month

The pre-MGYSS was 1.00 (1.00, 1.00) **(**Table 2**)**. One month and 3 months after surgery, the MGYSS was higher than the preoperative MGYSS, respectively (1.00 (1.00, 2.00) vs 1.00 (1.00, 1.00) and 1.00 (1.00, 2.00) vs 1.00 (1.00, 1.00)) **(**Table 2**)**. There was statistically significant difference between pre-MGYSS and MGYSS 3 months after surgery (*p* = 0.002), indicating that the MGYSS was worse after surgery.

The pre-CFS (CFS_0_) was 0.00 (0.00, 1.00) **(**Table 2**)**. The CFS at 1 month postoperatively (CFS_1_) was 1.00 (0.50, 1.00), and the CFS at 3 months postoperatively (CFS_3_) was 0.00 (0.00, 1.00) **(**Table 2**)**. There was significant difference between CFS_0_ and CFS_3_ (*p* = 0.008), suggesting that the CFS was aggravated after surgery.

The pre-TMH (TMH_0_) was 0.18 ± 0.03 mm **(**Table 2**)**. The TMH was 0.20 + 0.02 mm 1 month after surgery (TMH_1_), and the TMH was also 0.20 ± 0.02 mm 3 months after surgery (TMH_3_) **(**Table 2**)**. There were significant differences between TMH_0_ and TMH_1_ (*p* = 0.016), as well as between TMH_0_ and TMH_3_ (*p* = 0.020). Those results indicated that the TMH became better after surgery.

The pre-NITBUT (NITBUT_0_) was 5.52 ± 1.95 s **(**Table 2**)**. The TBUT was 5.06 ± 1.54 s 1 month after surgery (NITBUT_1_), and the TBUT was 4.99 ± 1.24 s 3 months after surgery (NITBUT_3_) **(**Table 2**)**. There were significant differences between NITBUT_0_ and NITBUT_1_ (*p* = 0.002), as well as between NITBUT_0_ and NITBUT_3_ (*p* = 0.035), which showed that the patient’s TBUT was shortened after surgery.

### Changes in DE syndrome and ocular surface parameters before and after cataract surgery in the OPT treatment group

The pre-OSDI score (OSDI_0_) was 31.39 ± 8.57, the OSDI score was 28.10 ± 5.88 months after surgery (OSDI_1_), and the OSDI score was 21.58 ± 4.97 3 months after surgery (OSDI_3_) **(**Table 3**)**. There were significant differences between OSDI_0_ and OSDI_1_ (*p* = 0.027), as well as between OSDI_0_ and OSDI_3_ (*p* = 0.000). Those results showed that after OPT treatment, the symptom of DE after surgery was not only ameliorated, but also superior to preoperative symptom.

**Table 3:** Comparison of dry eye symptoms and ocular surface parameters in the OPT treatment group before and after surgery in patients

parameters	baseline	1 month	3 month	*p* value		
				baseline vs 1 month	baseline vs 3 month	1 month vs 3 month
OSDI^a^	31.39 ± 8.57	28.10 ± 5.88	21.58 ± 4.97	0.027*	0.000*	0.000#
EMAS^b^	1.00 (1.00, 2.00)	1.00 (0.00, 1.25)	1.00 (0.00, 1.00)	0.020*	0.025*	0.739
MGYSS^b^	1.00 (1.00, 1.00)	1.00 (1.00, 1.00)	1.00 (0.00, 1.00)	0.414	0.020*	0.467
CFS^b^	0.00 (0.00, 1.00)	0.00 (0.00, 1.00)	0.00 (0.00, 1.00)	0.577	0.589	1.000
TMHa/mm	0.18 ± 0.31	0.19 ± 0.03	0.19 ± 0.02	0.210	0.147	0.611
NITBUT^a^/s	4.98 ± 1.84	5.67 ± 1.80	5.87 ± 1.17	0.091	0.026*	0.550
MGLS^b^	1.00 (1.00, 2.00)	1.00 (1.00, 2.00)	1.00 (1.00, 1.00)	0.083	0.002*	0.008#

*OSDI* Ocular Surface Disease Index, *MGYSS* Meibomian gland yielding secretion score, *CFS* corneal fluorescein staining, *TMH* tear meniscus height, *EMAS* Eyelid margin abnormality score, *MGLS* meibomian gland loss score, *TBUS* tear film break-up time. a: Normal distribution data, the mean is expressed as Mean ± SD, and the paired sample t test is used for comparison between groups. b: Non-normally distributed data, the mean is represented by Median (P25, P75), and the comparison between groups is based on paired sample nonparametric Wilcoxon test. *p < 0.05 vs Baseline; #*p* < 0.05 vs 1 month

The pre-EMAS (EMAS_0_) was 1.00 (1.00, 2.00), the EMAS was 1.00 (0.00, 1.25) 1 month after surgery (EMAS_1_), and the EMAS was 1.00 (0.00, 1.00) 3 months after surgery (EMAS_3_) **(**Table 3**)**. There were significant differences between EMAS_0_ and EMAS_1_ (*p* = 0.020), as well as between EMAS_0_ and EMAS_3_ (*p* = 0.025), which showed that after OPT treatment, the EMAS was improved.

The Pre-MGYSS was 1.00 (1.00, 1.00) **(**Table 3**)**. One month and 3 months after surgery, MGYSS were higher than preoperative MGYSS, respectively (1.00 (1.00, 1.00) vs 1.00 (1.00, 1.00) and 1.00 (0.00, 1.00) vs 1.00 (1.00, 1.00)) **(**Table 3**)**. The difference between preoperative MGYSS and MGYSS 3 months after surgery was statistically significant (*p* = 0.020), which suggested that after OPT treatment, the MGYSS was improved.

The pre-TBUT (NITBUT_0_) was 4.98 ± 1.84 s, the TBUT was 5.67 ± 1.80 s 1 month after surgery (NITBUT_1_), and the TBUT was 5.87 ± 1.17 s 3 months after surgery (NITBUT_3_) **(**Table 3**)**. There was significant difference between NITBUT_0_ and NITBUT_3_ (*p* = 0.026), which showed that after OPT treatment, the TBUT was ameliorated.

**Fig. 1:**
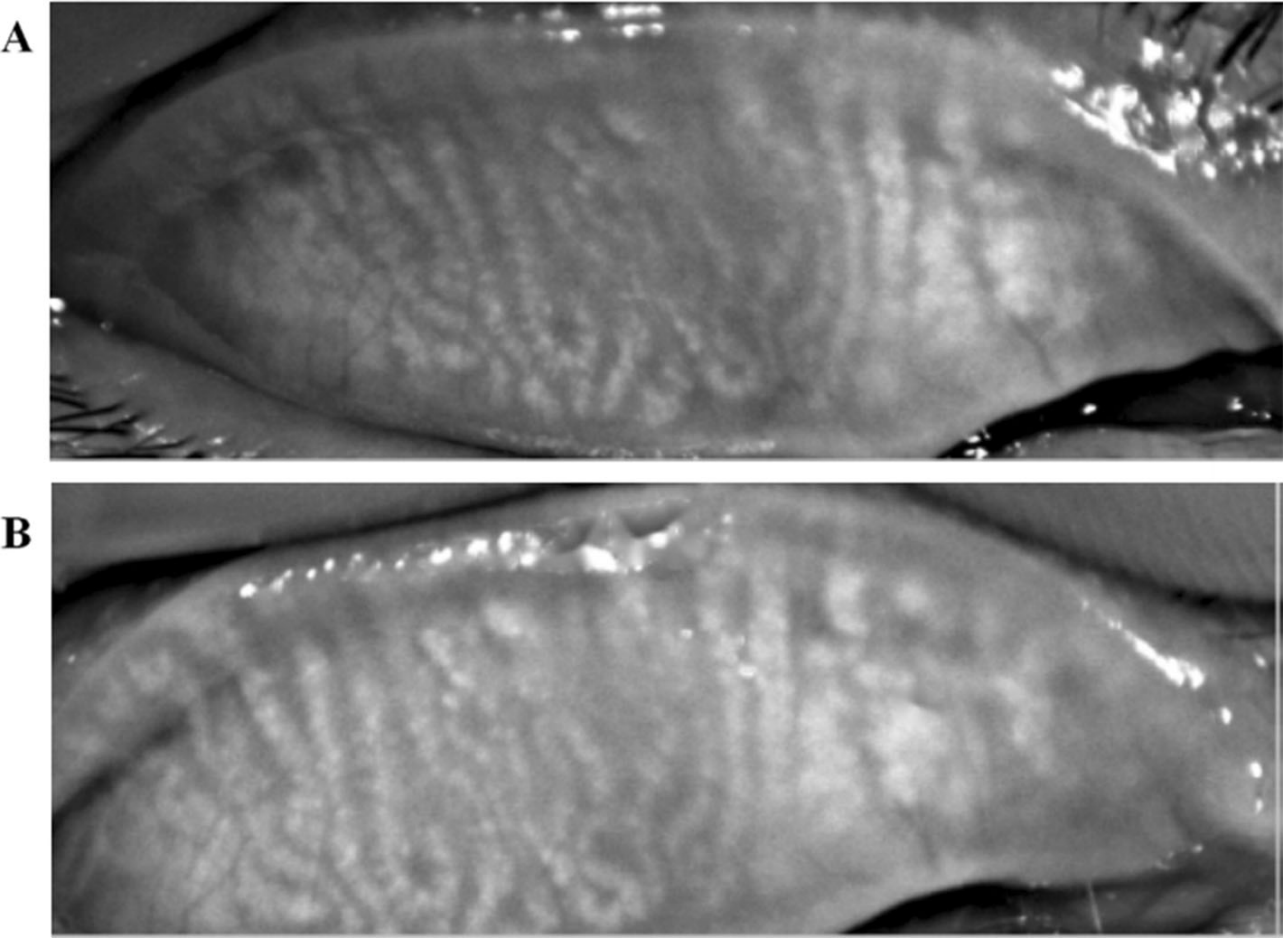
Meibography images. **a** Meibography image (100 × magnification) before OPT treatment. **b** Meibography image (100 × magnification) after OPT treatment. Compared the image before surgery, the MG structure of some patients was clear, and the loss rate was lower after OPT treatment

In the OPT treatment group, the MG structure of some patients was clear, and the loss rate was lower than that before surgery. The difference between the pre-MGLS and MGLS 3 months after surgery was statistically significant (*p* = 0.002) **(**Fig. 1**)**.

### Changes in ocular surface parameters between OPT treatment group and conventional operation group

**Fig. 2:**
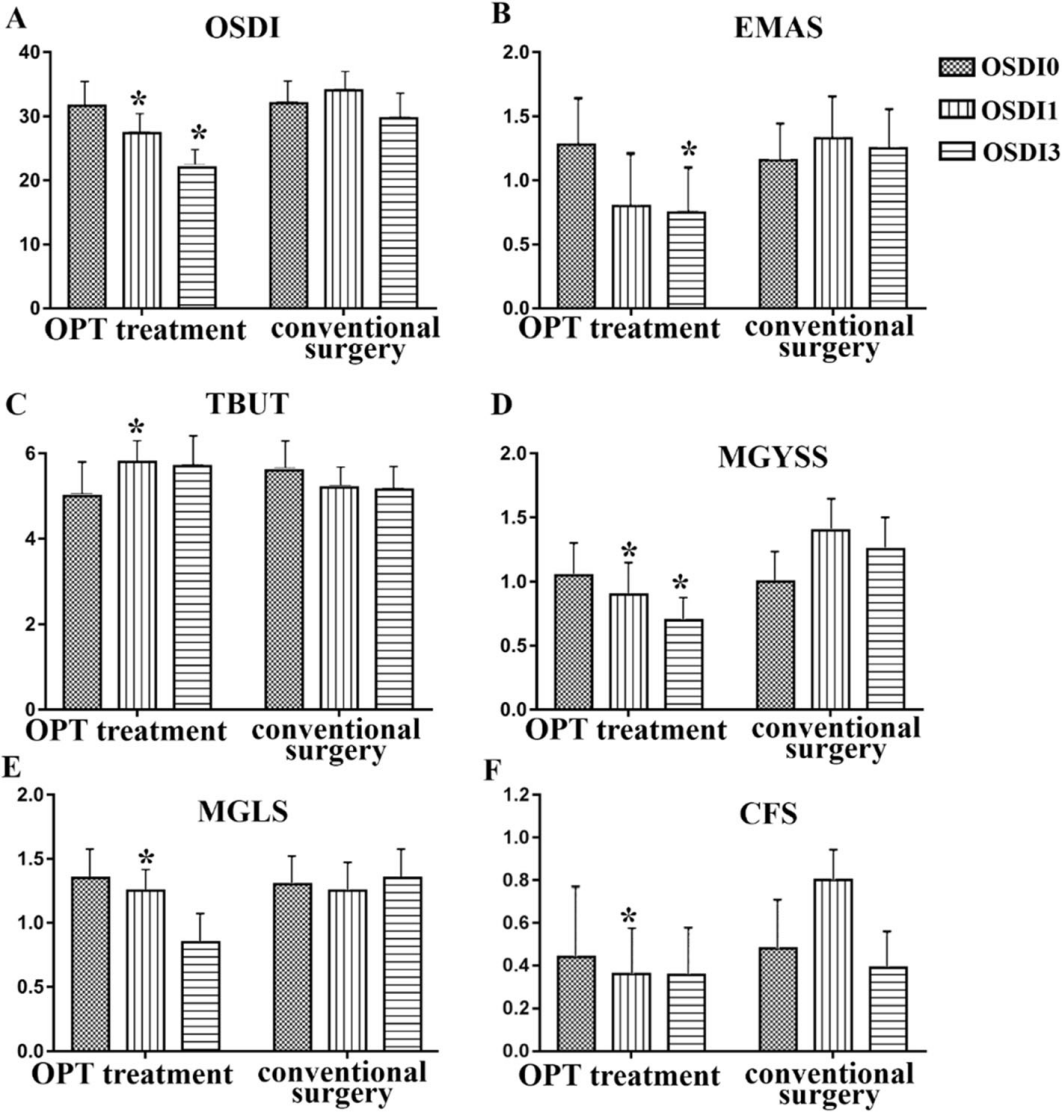
Changes in DE symptom and ocular surface parameters in the OPT treatment group and the conventional surgery group. **a** OSDI. **b** EMAS. **c** TBUT. **d** MGYSS. **e** MGLS. **f** CFS. *p < 0.05 vs the conventional surgery group

One month after surgery, there were notably significant differences in the scores of OSDI, EMAS, MGYSS and CFS between the conventional surgery group and the OPT treatment group (*p* < 0.05). In addition, 3 months after surgery, there were notably significant differences in the scores of OSDI, EMAS, MGYSS and CFS between the conventional surgery group and the OPT treatment group (*p* < 0.05). The postoperative comparison between the OPT treatment group and the conventional surgery group showed that the patients in the OPT treatment group had a better subjective feeling and ocular surface state after surgery **(**Table 4**,** Fig. 2**)**.

**Table 4:** Comparison of postoperative ocular surface parameters between OPT treatment group and Conventional operation group

parameters	*p* value		
	baseline	1 month	3 month
Age^a^	0.966		
OSDI^a^	0.931	0.005*	0.000*
EMAS^b^	0.543	0.060	0.033*
MGYSS^b^	0.657	0.004*	0.001*
CFS^b^	0.716	0.006*	0.800
TMH^a^/mm	0.416	0.189	0.110
NITBUT^a^/s	0.295	0.209	0.033*
MGLS^b^	0.544	0.989	0.005*

*OSDI* Ocular Surface Disease Index, *MGYSS* Meibomian gland yielding secretion score, *CFS* corneal fluorescein staining, *TMH* tear meniscus height, *EMAS* Eyelid margin abnormality score, *MGLS* meibomian gland loss score, *TBUS* tear film break-up time. a: Normal distribution data, the mean is expressed as Mean ± SD, and the paired sample t test is used for comparison between groups. b: Non-normally distributed data, the mean is represented by Median (P25, P75), and the comparison between groups is based on paired sample nonparametric Wilcoxon test. *p < 0.05 vs Baseline; #*p* < 0.05: conventional operation group vs OPT treatment group

## Discussion

MGD was divided into two major categories based on the secretion of Meibomian glands, namely low delivery and high delivery [23]. The low delivery type, including hypo secretory and obstructive, was the most common type of clinical MGD. Clinically, MGD is often associated with poor outcomes after cataract surgery, refractive surgery, and corneal surgery. Zhang et al. showed that patients with corneal epithelial erosion after cataract surgery combined with MGD may have had MGD before surgery [11]. MGD includes anatomical degeneration and pathophysiological changes, and clinicians and researchers agree that it seriously affects ocular surface health [24]. A large number of studies have shown that cataract surgery can aggravate MGD, resulting in lower satisfaction of patients with the surgical results [9–11, 22, 25]. In this study, there were significant differences in OSDI scores, MGYSS, TBUT, and CFS between the preoperative and postoperative outcomes. The results showed that cataract surgery can accelerate the development of MGD, which can cause dryness or increase the patient’s original dryness after surgery. However, there was no significant difference in the morphology and number of Meibomian glands before and after surgery in the conventional surgery group, suggesting that cataract surgery affected the Meibomian gland function of the patients, but did not change the anatomy of the meibomian gland. The purpose of treating MGD is to improve the secretion function of the meibomian glands, to improve the stability of the tear film, and to alleviate the symptoms of DE in patients.

The current treatment methods for MGD [26] include: (1) physical therapy: eyelid cleaning, hot compress, Meibomian Gland Expression (MGX), acupuncture, LipiFlow meibomian gland heat pulsation therapy, OPT treatment, and correcting the patient’s blinking habits; (2) drug treatment: artificial tears, non-steroidal anti-inflammatory drugs, antibiotics, hormone eye drops; (3) diet therapy: omega-3 fatty acids. Although there are many ways to treat MGD, there is currently no definitive and effective treatment for MGD. Besides, many treatments cannot be adhered to because of their poor compliance. In-tense pulsed light (IPL) was first reported for the treatment of MGD in 2015 and then there were several studies to report its efficacy in the treatment of MGD. The M22 system uses Optimized Pulsed Technology (OPT), which has a uniform square wave pulse waveform, and the time and energy of intense light emission are more accurate, safe, and effective.

In this study, OPT treatment was better in improving OSDI, TBUT and MG functions. There were significant differences in OSDI, EMAS, MGYSS and CFS 1 month and 3 months after surgery. No complications such as iris depigmentation and dilated pupils appeared during treatment, which indicated the efficacy and safety of OPT treatment. The results of this study are consistent with those of previous studies [27–29].

In previous studies, it was often combined with MGX immediately after OPT treatment, because researchers considered that the thermal effects of OPT may make meibum easy to discharge. However, in this study, patients who underwent OPT treatment did not undergo MGX because there were no high-restorative patients enrolled in this study. The mechanism of OPT treatment for MGD may be the following [30]: (1) thermal effects improved glandular secretion and excretion; (2) inflammatory response and edema of acinar were reduced by blocking dilated capillaries and reducing inflammatory mediators release; (3) the load of bacteria and aphids were decreased. Yin [31] confirmed that OPT not only improved the macrostructure of MG but also changed the microstructure of MG, which suggested that the light simulation mechanism, anti-inflammatory mechanism and photothermic effect were the main mechanisms of OPT treatment for MGD.

Simple eyelid cleaning, hot compress or combined MGX can improve the function of meibomian glands [32, 33]. Sravanthi Vegunta and other researchers have reported that IPL and MGX can significantly improve 89% DE symptoms and 77% meibomian gland function in patients [18]. Dell’s study confirmed that the combination of OPT and MGX was effective in relieving the symptoms and signs of patients with evaporative dry eye secondary to MGD [34].

This study also has some limitations. Firstly, this study was conducted in a relatively small number of subjects. Secondly, the meibomian gland discharge capacity was not scored due to the absence of meibomian gland evaluator. Thirdly, the OPT did not directly act on the upper and lower eyelids. It was reported that direct OPT treatment in upper and lower eyelids would bring more evident effect [30]. In this study, both eyes were treated with OPT at the same time and the range of energy we selected was higher than that reported in previous studies, which may be the reasons for the significant effect of OPT treatment in this study.

Cataract surgery for patients with Age-related cataract is not only for the simple improvement of visual acuity, but also in significantly improving patients’ visual quality and even living quality. Therefore, the ophthalmologist is required to carefully evaluate the patient’s ocular surface state before surgery, especially for patients with MGD, to improve the satisfaction of patients with MGD in cataract surgery. The ophthalmologist should also educate and intervene the patients before surgery, operate carefully during operation, use drugs rationally after surgery and quest the individualized management mode of patients with different degrees of ocular surface diseases.

## Conclusions

In conclusion, our study suggested that phacoemulsification can increase the DE symptoms and MGD. OPT treatment was a safe and effective intervention for patients with cataract and MGD during the perioperative period.

## Data Availability

Not applicable.

## Abbreviations

ARC: Age-related cataract
CFS: Corneal fluorescein staining
DE: Dry eye
EMAS: Eyelid margin abnormality score
IPL: In-tense pulsed light
MGD: Meibomian gland dysfunction
MGX: Meibomian Gland Expression
MGYSS: Meibomian gland yielding secretion score
OPT: Optimal Pulsed Technology
OSDI: Ocular Surface Disease Index
TBUT: Tear film break-up time
TMH: Tear meniscus height
